# Toxicogenomics of the Freshwater Oligochaete, *Tubifex tubifex* (*Annelida*, *Clitellata*), in Acute Water-Only Exposure to Arsenic

**DOI:** 10.3390/ijms25063382

**Published:** 2024-03-16

**Authors:** Iñigo Moreno-Ocio, Mónica Aquilino, Lola Llorente, Maite Martínez-Madrid, Pilar Rodríguez, Leire Méndez-Fernández, Rosario Planelló

**Affiliations:** 1 Department of Zoology and Animal Cell Biology, University of the Basque Country (UPV/EHU), 48940 Leioa, Spain; inigo.moreno@ehu.eus (I.M.-O.); pilar.rodriguez@ehu.eus (P.R.); 2 Molecular Entomology, Biomarkers and Environmental Stress Group, Faculty of Science, Universidad Nacional de Educación a Distancia (UNED), 28232 Las Rozas de Madrid, Spain; maquilino@ccia.uned.es (M.A.); lola13llorente@gmail.com (L.L.); 3 Department of Genetics, Physical Anthropology and Animal Physiology, University of the Basque Country (UPV/EHU), 48940 Leioa, Spain; maite.martinez@ehu.eus

**Keywords:** arsenic, oligochaeta, toxicity assay, transcriptional biomarker, cell stress response, oxidative stress, homeostasis, effect concentration, reference transcriptome

## Abstract

A toxicogenomic approach was used for toxicity evaluation of arsenic in the aquatic environment, and differential gene expression was investigated from 24 h and 96 h water-only acute toxicity tests with the aquatic oligochaete, *Tubifex tubifex* (*Annelida*, *Clitellata*). Several toxicological endpoints (survival and autotomy) of the oligochaete and tissue residues were measured, and dose-response modelling of gene expression data was studied. A reference transcriptome of the aquatic oligochaete, *T. tubifex*, was reconstructed for the first time, and genes related to cell stress response (*Hsc70*, *Hsp10*, *Hsp60*, and *Hsp83*), energy metabolism (*COX1*), oxidative stress (*Cat*, *GSR*, and *MnSOD*), and the genes involved in the homeostasis of organisms (*CaM*, *RpS13*, and *UBE2*) were identified and characterised. The potential use of the genes identified for risk assessment in freshwater ecosystems as early biomarkers of arsenic toxicity is discussed.

## 1. Introduction

Arsenic (As) is a common metalloid that occurs in the air, soil, water, and all living tissues [1] and is one of the ubiquitous metalloids of widespread public concern. It is present in the environment from both natural and anthropogenic sources. One of the main sources of arsenic and other metal local contamination in freshwater ecosystems are past and present mining activities [2,3,4]. In fact, arsenic appears in high concentrations in groundwater from geological sources, causing chronic health disorders [5]. Arsenic pollution in sediments and bioaccumulation has been demonstrated as one of the main causes of toxicity [3], and of the alteration of the ecological status in rivers affected by mining activities [6,7]. Integrating chemical analysis with biological endpoints can help in the monitoring and protection of aquatic ecosystems. In this sense, molecular, biochemical, and physiological biomarkers are useful tools to assess the impact of environmental contaminants on species’ health and identify those at risk [8,9]. For instance, alterations in the expression levels of the relevant genes have been described as good biomarkers after exposure to heavy metals, UV filters, phthalates, or pesticides, and among others, those related to the stress response (e.g., *Hsps*), the hormonal system pathways (e.g., *EcR*), the energy metabolism (*COX1*, *gapdh*), and the biotransformation and detoxification processes (e.g., *Cyp450*, *CAT*, *GPx*, *GST*, *SOD*) [8,9].

Even though the mechanisms of arsenic toxicity are not yet fully understood, oxidative stress is considered one of the major processes in arsenic-mediated toxicity. Thus, biomarkers of oxidative damage and antioxidant enzymes have been extensively used to assess the ecotoxicity of contamination [10]. The generation of reactive oxygen species (ROS) and the occurrence of oxidative DNA damage have been accepted because of arsenic exposure and are proposed as two of the main mechanisms of the action of arsenic carcinogenicity [11]. Furthermore, the production of methylated arsenic species leads to several epigenetic and genetic alterations that culminate in DNA damage [11].

In the present study, we used the worm, *Tubifex tubifex* (*Annelida*, *Clitellata*), a typical benthic invertebrate with widespread distribution and a good tolerance to some stress factors (e.g., pollution), resulting as being an important bioindicator species of interstitial benthic fauna [12]. The physiological and metabolic activities of *T. tubifex* are substantially mediated by multiple environmental factors such as hydrodynamics [13], temperature [14], and the presence of pollutants [15,16,17].

*T. tubifex* is widely distributed, abundant in temperate regions, and easy to culture under laboratory conditions. It has been used in toxicity studies as a test organism for a long time [18], its relevance has been pointed out by Chapman [19], and there are standardised test methods that include this species to study the effects of hazardous chemicals [20,21], among others, toxicity tests for As, in both water-only and sediment exposures [22,23].

To date, there is scarce information available on this species and other aquatic oligochaetes in genomic databases, and most data refer to studies with earthworms (e.g., [24,25]). This makes it difficult to decipher the mechanism of toxic action of this compound at the early molecular level (gene expression).

Therefore, the aim of this study was: (1) to assess the genetic mechanisms of action of As in relation to exposures producing an acute toxic effect; (2) to evaluate the importance of *T. tubifex* as a model species for assessing the toxicogenomics of As in aquatic environments; (3) to study the gene expression in relevant routes related to the survival of the worms in response to As. To this end, we identified and characterised de novo in *T. tubifex*, genes related to cell stress response, energy production, detoxification and oxidative stress, and the genes involved in the homeostasis of organisms; secondly, we analysed the transcriptional activity of these genes after exposure to arsenic stress. This work was conducted to describe potential biomarkers of toxicity and to assess their usefulness as early biomarkers of arsenic effect in this species.

## 2. Results

### 2.1. Water Acute Toxicity Test for As

The 96 h water-only toxicity test was validated based on the physicochemical conditions which ranged within values indicated by the American Society for Testing and Materials [20]; in the overlying water, pH values were 6–9, and dissolved oxygen concentration was kept above 2.5 mg L^−1^ (Table A1). The control batch showed 96% survival, and autotomy of the posterior body region was not observed. No other effects were recorded in control organisms.

The raw results of the acute toxicity test are shown in Table 1. Regarding mortality, after 24 h exposure it was only observed at the three highest concentrations (159, 250, and 407 mg As L^−1^); 96 h exposures led to a higher mortality for all As concentrations except the two lowest concentrations (38 and 63 mg As L^−1^).

The concentration of As related to 50% mortality and autotomy was given by the parameters LC_50_ and EC_50_, respectively (Table 2). The As LC_50_ was 136.87 mg L^−1^ at the end of 96 h exposure, and As EC_50_ levels were slightly lower regarding autotomy.

Table 3 shows the As tissue concentrations related to 50% of the maximum mortality (LR_50_) and autotomy (ER_50_). The LR_50_ was 1072 µg As g^−1^ dw in the first 24 h of exposure (the maximum tissue residue obtained in the experiment was 1083 µg g^−1^ dw); and this concentration decreased with time until 578 µg g^−1^ dw at 96 h (CI95% = 574–582). The tissue concentration related to autotomy was 479 µg g^−1^ dw at 96 h (CI95% = 477–481), slightly lower than mortality.

### 2.2. Reference Transcriptome

An extensive transcriptome of *T. tubifex* was obtained from worms after exposure to As. Data were deposited in the European Nucleotide Archive (ENA) at EMBL-EBI under accession number PRJEB50858. The assembly of the high-quality reads reported a total of 107,774 Clustered Contig ‘Unigenes’ with an N50 length of 810 bp and 34,331 predicted ORFs. The number of successfully annotated contigs was 24,806. Among them, the GO categorisation identified 67 GO Terms, within three categories: biological process (33), cellular component (19), and molecular function (15).

### 2.3. Gene Characterisation

**Figure 1:**
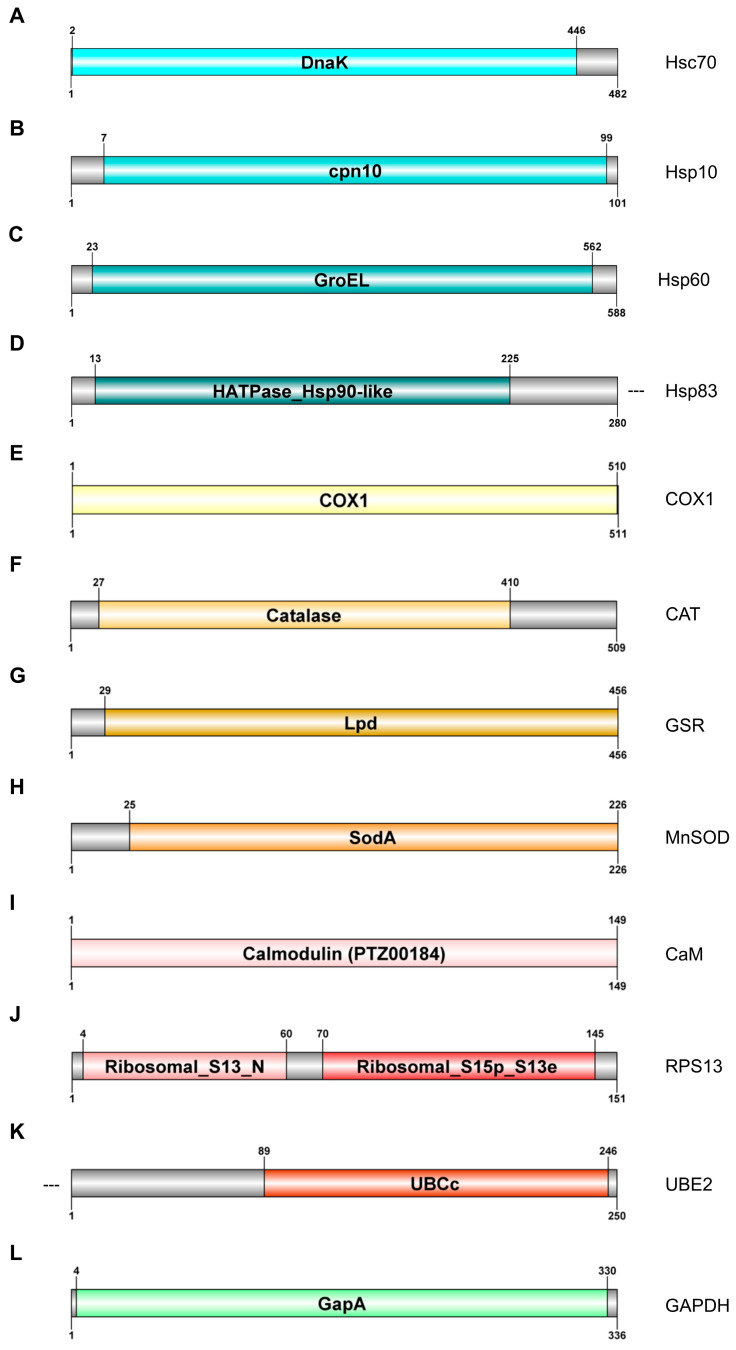
Characterisation of proteins identified in the de novo transcriptome of *T. tubifex*. Diagram of the protein of *T. tubifex* identified as putative mRNAs and their conserved domains: Hsc70 (**A**), Hsp10 (**B**), Hsp60 (**C**), Hsp83 (**D**), COX1 (**E**), CAT (**F**), GSR (**G**), MnSOD (**H**), CaM (**I**) RPS13 (**J**), UBE2 (**K**) and GAPDH (**L**). Diagram designed with DOG V.2 software. (- - -) means that this protein is incomplete in one or other end of the protein.

Nine sequences with the complete ORF (*Hsc70*, *Hsp10*, *Hsp60*, *COX1*, *Cat*, *GSR*, *MnSOD*, *CaM*, and *RpS13*) and two incomplete sequences (*Hsp83* and *UBE2*) were obtained. Two reference genes were also identified, the complete ORF of *Gapdh* and a fragment of *28S* rRNA. Their sequences were registered in the GenBank database as PP213149 (*Hsc70*), PP213150 (*Hsp10*), PP213151 (*Hsp60*), PP213152 (*Hsp83*), PP213153 (*Cat*), PP212898 (*COX1*), PP213154 (*GSR*), PP213155 (*MnSOD*), PP213156 (*CaM*), PP213157 (*RpS13*), PP213158 (*UBE2*), and PP213159 (*Gapdh*). Relevant domains of each ORF are presented in Figure 1.

#### 2.3.1. Cell Stress Response Genes

Four nucleotide sequences encoding different heat shock proteins were identified (Figure 1A–D).

The complete ORF of the *Hsc70* gene was 1449 bp. It encoded a 482 aa sequence which contains the molecular chaperonin DnaK (HSP70) domain (Figure 1A). It shared 87% identity with *Biomphalaria glabrata* and *Sacostrea echinata*.

The complete ORF of the *Hsp10* gene was 306 bp in length; it encoded a 101 aa protein with a chaperonin 10 Kd subunit domain covering nearly all the protein, and it shared 76% identity with the *Crassostrea gigas* and 75% identity with *Physella acuta* (Figure 1B).

The second complete ORF corresponded to the *Hsp60* gene (Figure 1C), a 1767 bp sequence encoding a protein with 588 residues and a highly conserved GroEL heat shock protein 60 domain. It shared 81% identity with *Eisenia fetida* and 80% identity with diverse *Helobdella robusta*.

Finally, an incomplete ORF of 840 pb encoded a 280 aa sequence, corresponding to the 5′ end of the heat shock protein 83 (Hsp83) (Figure 1D). It contains a specific HATPase_Hsp90-like domain (aa 13–225) and a specific HATPase_c domain (aa 35–168). It shared 87% identity with *Halyomorpha halys* and 82% with *Arma chinensis*.

#### 2.3.2. Energy Metabolism and Oxidative Stress Response

The complete ORF of cytochrome c oxidase I gene (*COX1*) was 1536 bp in length, encoding a 511 aa protein that includes a cytochrome c oxidase I domain (COX1; aa 1–510). This protein shared 91% identity with other *T. tubifex* and 81% with *Nais communis* (Figure 1E).

The *Cat* ORF (1530 bp) encoded a 509 aa protein with a conserved catalase domain from residues 27 to 410 (Figure 1E). The protein shared more than 70% identity with catalase genes from annelid species such as *E. fetida* (74%) or *Haliotis madaka* (74%) (Figure 1F).

The complete ORF of glutathione reductase gene (*GSR*) was 1371 bp in length; it encoded a protein of 456 aa that contained a pyruvate/2-oxoglutarate complex (Lpd specific domain), characteristic of glutathione reductases (Figure 1H). This protein shared 65% identity with *Haliotis fulgens* and *Pomacea canaliculata* (Figure 1G).

Finally, the complete ORF of manganese superoxide dismutase gene (*MnSOD***)** was 681 bp long and encoded a 226 aa protein with a superoxide dismutase (SodA) specific domain (aa 25–226); it shared 69% identity with both *Eisenia andrei* and *Zootermopsis nevadensis*) (Figure 1H).

#### 2.3.3. Homeostasis Maintenance 

The complete ORF of the *CaM* gene was 450 bp encoding a protein with 149 aa corresponding entirely to a calmodulin (PTZ00184) domain (Figure 1I); it shared 100% and 99% identity with *Lumbricus rubellus* and *Pinctada fucata*, respectively.

The complete ORF of 40S ribosomal protein S13 (*RpS13*) was identified; it was 456 bp in length and encoded a protein with 151 aa residues with two specific domains: ribosomal_S13_N and Ribosomal_S15p_S13e; it shared 91% identity with *Arenicola marina* and 88% with *Lumbricus rubellus*.

Finally, incomplete ORF of the ubiquitin-conjugating enzyme E2 gene (*UBE2*) had 755 bp in length and covered a region of 250 aa of the C-terminal. It had a ubiquitin-conjugating enzyme E2 catalytic domain (UBCc; aa 89–246), sharing 65% identity with *Branchiostoma floridae* and 63% with *Lingula anatine*) (Figure 1K).

#### 2.3.4. Reference Genes

The complete ORF of the *Gapdh* gene was 1011 bp long and coded for a 336 aa protein containing a conserved domain covering most of the protein (GapA; aa 4–330). This protein showed 81% identity *Enchytraeus albidus* and 78% with *Mesenchytraeus hydrius*.

### 2.4. Gene Expression Study

**Figure 2:**
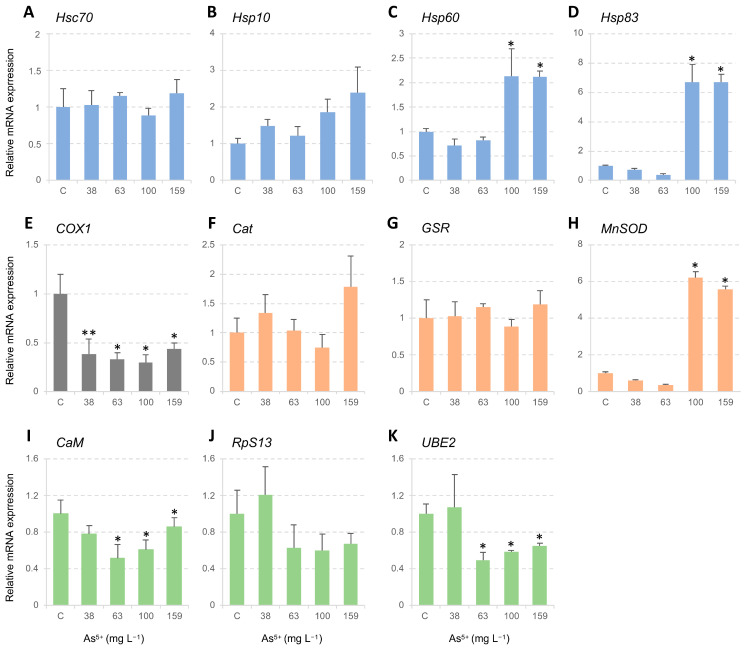
Transcriptional activity of genes related to cell stress response, oxidative stress response, and homeostasis in *T. tubifex* exposed for 96 h to 38 mg L^−1^, 63 mg L^−1^, 100 mg L^−1^, and 159 mg L^−1^ arsenic. Error bars indicate SE. Normalisation was performed using *Gapdh* and *26S* as the reference genes. Bars represent the relative mRNA expression of *Hsc70* (**A**), *Hsp10* (**B**), *Hsp60* (**C**) and *Hsp83* (**D**), *COX1* (**E**), *Cat* (**F**), *GSR* (**G**) and *MnSOD* (**H**), *CaM* (**I**), *RpS13* (**J**), and *UBE2* (**K**) measured using real-time RT-PCR. Asterisks indicate significant differences with respect to control (**C**) values: *p* < 0.05 (*); *p* < 0.1 (**).

The transcriptional profile of different genes of interest under the selected experimental conditions were analysed through quantitative real-time PCR. For this purpose, *T. tubifex* adults exposed to 38, 63, 100, and 159 mg L^−1^ As in acute (after 96-h exposure) toxicity studies were used (Figure 2).

#### 2.4.1. Cell Stress Response 

From the four molecular cell stress-involved analysed genes, the most striking differences compared to control values were those related to *Hsp83* and *Hsp60*, as arsenic exposure led to a significant induction of transcriptional expression of both genes after the highest doses (Figure 2C,D). The upregulation was stronger for *Hsp83*, by up to 7.3- and 6.7-fold after 100 mg L^−1^ and 159 mg L^−1^, respectively (*p* < 0.05). Regarding the inducible mitochondrial HSP10, transcript levels of the *Hsp10* gene triggered a trend to increase, especially after 100 mg L^−1^ and 159 mg L^−1^ (up to 1.8- and 2.4-fold above control values, respectively), although with no statistical significance (Figure 2B). Finally, as expected, as a constitutive protein, *Hsc70* gene activity remained stable after arsenic exposure (Figure 2A).

#### 2.4.2. Energy Metabolism and Oxidative Stress Biomarkers 

The most acute statistically significant alterations were found for *COX1* and *MnSOD* transcript levels after 96 h exposure in a different manner. The expression of *COX1* dropped 61% and 45%, respectively, below control values (Figure 2E). In contrast, a clear time-dependent upregulation was observed for *MnSOD* with statistically significant changes after 96 h exposure to 100 and 159 mg L^−1^ As with respect to control values (Figure 2H). No significant changes were detected in the other two examined genes, *Cat* and *GSR*, after As exposure, compared to control conditions. The same tendency was observed in both genes, with a slight induction at 38, 63, and 159 mg L^−1^, and with values of expression below control values at 100 mg L^−1^ As (ns; *p* > 0.05) (Figure 2F,G).

#### 2.4.3. Transcriptional Alterations in Homeostasis-Related Biomarkers 

A dose-dependent repression of the *CaM* gene was observed after exposure to As, by 29% at 63 mg L^−1^, 40% at 100 mg L^−1^, and 23% at 159 mg L^−1^ compared to control values (*p* < 0.05) (Figure 2I). A similar response was detected in the *UBE2* gene, whose expression fell by 51%, 42%, and 36%, respectively, below control values (Figure 2K). Finally, although not significant, gene coding to small ribosomal protein RPS13 was also dose-dependent downregulated with values of about 33–40% below control values, under the highest concentration of arsenic tested (Figure 2J).

## 3. Discussion

This work describes for the first time *T. tubifex* genes related to cell stress response, energy metabolism, oxidative stress response, and homeostasis maintenance response. Using the information from our transcriptome on databases (Ref. PRJEB50858), the genes *Hsc70*, *Hsp10*, *Hsp60*, *Hsp83*, *COX1*, *Cat*, *GSR*, *MnSOD*, *RpS13*, and *UBE2* were identified and characterised. We used these potential biomarkers to analyse and compare, at molecular level, the transcriptional responses of different routes. From a general perspective, the impact of Arsenic caused an upregulation of the cell stress and oxidative stress response. This kind of response was previously described under exposure to cationic and anionic surfactants that modulate the levels of oxidative stress enzymes and energy metabolism in *T. tubifex* [26,27].

Soil and groundwater contamination with this metalloid has become a major concern in areas where there are tailings and wastes from mining activities. Arsenic contamination, particularly in groundwater, has already been reported in almost all continents of the world [28], recognised as a toxic metalloid and severe threat to biodiversity due to its contamination in freshwater ecosystems, such as death and malformations of toad embryos, ability to reduce the reproductive capacity in copepods growth inhibition of algae, or mortality of amphipods, gastropods, and copepods [29,30], and growth inhibition of *C. elegans* [31].

Previous studies on the water-only acute toxicity of *T. tubifex* exposed to As reported comparable results to the present study. Thus, 96 h-LC_50_ estimated by Fargasova, 1994 [32] and Lobo et al., 2016 [22] were 127 mg L^−1^ and >118.18 mg L^−1^, respectively. The EC_50_ estimated here for autotomy were very similar, although slightly lower, than those estimated from lethal effects. On the contrary, [33] proposed a LR_50_ that is much lower (8.9 mg L^−1^), compared with the above-mentioned data. These discrepancies could be a result of the sensitivity of the test organisms (e.g., age or culture characteristics), but may also be due to the use of different test conditions (30 °C). In fact, metal toxicity has been demonstrated to increase with temperature [34,35,36].

There are no previous studies on As-effective tissue residues in *T. tubifex* from acute toxicity tests for comparison. In the present study, differences between estimated LR_50_ and ER_50_ for mortality and autotomy were greater with exposure time (95% confidence limits did not overlap at 72 h and 96 h of exposure). It is interesting to note that the LR_50_ and ER_50_ are comparable with those estimated in *T. tubifex* from the 28-day sediment toxicity test (1002 ± 55 μg g^−1^ dw: [23]; 1191.2 ± 151.3 μg g^−1^ dw: [3]). These results demonstrate that the LR_50_ and ER_50_ calculated from acute water-only exposure can give, with caution, a good approximation to the tissue residues after sediment chronic exposure. The results are also comparable with a field approach for the estimation of tissue residues of ER_50_ for aquatic Microdrile oligochaetes (ER_50_ = 509–570 μg g^−1^ dw: [7]) related to the 50% reduction in the river macroinvertebrate ecological status. Interestingly, that range of ER_50_ values from field aquatic oligochaete worms overlaps, or is very close to, the ER_50_ values estimated in the present study for mortality and autotomy after 72 h and 96 h exposure. This suggests that the molecular information studied could be expected to also be useful in a more realistic scenario, with field aquatic oligochaetes as bioindicators of As pollution.

The analysis of genomic information enables the identification of novel target genes to deepen the knowledge of the effects of pollutants in exposed biota. This traditionally occurs through genomic, transcriptomic, and metabolomic analyses of model organisms; however, there is little genomic information available for many, including the freshwater oligochaete, *T. tubifex*.

In general, As toxicity mechanisms include oxidative stress, excessive ROS production, and changes in signalling pathways [37], as well changes in gene expression [38,39]. Furthermore, it has been shown that As can cause toxicity in cells or tissues by increasing the generation of ROS and stimulating the damage of essential cellular biomolecules such as DNA, lipid, and proteins [40,41]. The toxic effects of acute arsenic treatment have also been described on the behaviour, cognition, and development of *Drosophila melanogaster* [42].

In this work, we built a de novo transcriptome (Ref. PRJEB50858) of individuals of *T. tubifex* from a laboratory culture (UPV/EHU, Bilbao, Spain). In addition, to assess their response as biomarkers of exposure to As, we identified and characterised for the first time in this species, genes related to cell stress response (*Hsc70*, *Hsp10*, *Hsp60*, *Hsp83*), oxidative stress response (*Cat*, *GSR*, *MnSOD*), the energy metabolism (*COX1*), and genes involved in the homeostasis of organisms (*CaM*, *RpS13*, *UBE2*). These data provide the scientific community with new potential biomarkers that could help in the assessment of the early toxic effects of environmental stressors.

Our bioassays proved the impact of arsenic in terms of transcriptional alterations in *T. tubifex*. The exposure to As resulted in the overexpression of cell stress response markers (i.e., *Hsp83*, *Hsp60*), the alteration of the oxidative stress response *(MnSOD*), and the mitochondrial metabolism (*COX1*) also in relevant genes for the maintenance of homeostasis (*UBE2*, *CaM*, *RpS13*). Our results are in accordance with the induction of *mtl* and oxidative stress response (*cyp-35A2*, *sod-1*) in *Lysinibacillus sphaericus* [43] and the upregulation of genes involved in the oxidative stress response and effects of genes related with epigenetic markers described in *Chironomus riparius* [44]. Other heavy metals, such as Cd and Hg, deregulate the genes involved in oxidative stress, which contrasts with the results obtained in this study and points to a heavy metal differential response in this essential pathway [45]. Significant alterations were also observed in the enzymatic activities of catalase (CAT), glutathione peroxidase (GPx), glutathione S-transferase (GST), and superoxide dismutase (SOD), in *Chironomus dilutus* exposed to sediments containing complex mixtures of metals and arsenic [46].

Two HSP genes (*Hsp83* and *Hsp60*) presented weak variations in the level of messenger after 96 h of exposure to 100 mg L^−1^ and 159 mg L^−1^ of As. An increase in the quantity of HSP proteins after metal exposure has been previously described in *Lumbricus terrestris* and *E. fetida* [47,48]. The activity of HSP genes was also altered by cadmium in *Protohermes costalis* (Megaloptera) [49] and other aquatic organisms [50]. Hence, our results reinforce the idea of HSPs being suitable biomarkers of metal exposure.

Cytochrome c oxidase is the terminal electron acceptor of the mitochondrial electron transport chain, catalysing the oxidation of ferricytochrome c [51]. Cytochrome oxidase, an iron-containing enzyme, is a crucial component of oxidative phosphorylation, which leads to the generation of aerobic energy. This enzyme acts in the mitochondrial electron transport chain, to finally convert catabolic glucose products into adenosine triphosphate (ATP). Our results showed the dose-dependent repression of *COX1* transcriptional activity of up to 63%, 62%, and 52% below control values, after exposure to 63, 100, and 159 mg L^−1^ of As, respectively. The inhibition of COX1 enzyme activity was previously described for other metals such as Cd^2+^ and Zn^2+^ in *Rhodobacter sphaeroides*, leading to a slow and less efficient process of proton pumping [52]. The impact of the inhibition of *COX1* might include an energy crisis due to lower ATP production, and increased formation of ROS in mitochondria, as described by Srinivasan and Avadhani [51]. Arsenite was also shown to diminish the function of the electron transport chain, disrupting the pyruvate metabolism in nematodes and human cells [53,54]. Our data suggest that arsenic could repress proton movement through a proton exit path, which can impair, in terms of regulation, the efficiency of energy transduction in mitochondria.

As mentioned above, antioxidant enzymes are explicit biomarkers of oxidative stress that can decimate ROS species and other cell pro-oxidative enzymes [55]. The oxidative stress enzyme which provides a major defence against oxidative stress, by converting reactive oxygen radicals to H_2_O_2_ through the process of dismutation, is superoxide dismutase (SOD) [56,57]. SOD helps to facilitate the transformation of superoxide anion radicals to hydrogen peroxide (H_2_O_2_). In the present study, *SOD* activity increased significantly by 6-fold and 5-fold after 96 h in the concentrations of As (100 mg L^−1^ and 159 mg L^−1^, respectively) as compared to the control. This spike in *SOD* activity could be due to superoxide ion induction, which activates the biosynthesis of SOD, bulwarking cells against oxidative damage, as described in *E. fetida* [58]. SOD activity levels also increased in *T. tubifex* worms exposed to HgCl_2_ [59]. The increased SOD activity in the *T. tubifex* might be a compensation mechanism against arsenic intoxication.

Calmodulin (CaM) is a versatile Ca^2+^-sensor/transducer protein that modulates hundreds of enzymes, channels, transport systems, transcription factors, adaptors, and other structural proteins, controlling multiple cellular functions [60]. Our work described a significant dose-dependent repression of *CaM* after exposure to 63, 100, and 159 mg L^−1^ of As. Ca^2+^/CaM is responsible for the activation of CaMKII, the most abundant multifunctional protein in neurons, which initiates the biochemical cascade, thereby facilitating synaptic transmission and synaptic plasticity. Arsenic in astrocytes might impair synaptic formation through disturbing astrocytic effects on neuronal signal transduction [61]. The expression of CaMKII was decreased in mice exposed to arsenic, leading to the induction of damage in the structure of the hippocampus and to impairment of learning and memory [62]. According to that, our data suggest that oxidative stress might perturb neuronal function via decreased calmodulin levels and consequently, the ability to bind, activate, and regulate the interactions of CaMKII [63].

The ubiquitin-proteasome pathway (UPP) is the central protein degradation system in eukaryotic cells, playing a key role in homeostasis maintenance through the proteolysis of regulatory as well as misfolded (potentially harmful) proteins [64]. For its purpose, the UPP intersects many cellular events, such as cell cycle, apoptosis, cell survival, and DNA repair [65]. The inhibition of proteasomal activity causes inclusion formation in neuronal and non-neuronal cells related to neurodegenerative diseases [66]. Although the molecular mechanism is unclear, there is strong evidence that implicates abnormal processing of a variety of cellular proteins via the ubiquitin/26S proteasome system in neurodegenerative disease development. The *UBE2* repression observed after arsenic exposure in *T. tubifex* suggests that this compound might impair the UPP normal activity and, therefore, perturb neuronal function.

To date, only a few studies in invertebrates have focused on the nucleolar region as a potential target of exposure to xenobiotics, and data regarding invertebrates are particularly scarce. In *C. riparius* larvae, cadmium exposures led to a significant reduction in the 32S and the 45S rRNA precursors [67]. Additionally, the repression of ribosomal protein genes was also reported in *C. riparius* exposed to cadmium and silver nanoparticles [68], and after acute 24 h and 48 h exposure to a wide range of DEHP doses [69]. Although not significant, our work shows a dose-dependent trend towards *RpS13* repression. An in-depth study of the nucleolar region in *T. tubifex* and the effects that arsenic might have on it, is still necessary to decipher the potential effects of this heavy metal on the ribosomal machinery synthesis. Our results clearly demonstrate the usefulness of implementing molecular toxicity studies in laboratory populations of *T. tubifex* to define, with more precision, the targets of the effects that are altered by exposure to different heavy metals. The next step in our study will be to investigate new biomarker genes related to other relevant metabolic pathways to deepen our understanding of the physiological response of *T. tubifex* to pollutants. For instance, our objective is to assess in this species the toxicity of field-collected sediments from different mining areas, exhibiting a gradient of heavy metal contamination (i.e., Cd, Pb, etc.), in order to evaluate the type of responses that are triggered depending on the circumstances of a real scenario, in which worms are chronically exposed to complex mixtures of pollutants.

This will broaden the practical applicability of this organism to evaluate the degree of contamination over a wider range of freshwater ecosystems and will add relevant knowledge on the different biochemical routes affected by different pollutants.

## 4. Materials and Methods

### 4.1. Water-Only Acute Toxicity Test for As

An acute water-only toxicity test was run using the oligochaete, *T. tubifex*, from the laboratory culture stock from the University of the Basque Country (UPV/EHU); the specimens had an age of 5 weeks old and were immature. The culture was kept at 22 ± 1 °C, in the dark, with gentle aeration, and with about a 3 cm sediment layer, sieved through a 250 μm mesh size, covered by dechlorinated tap water and a food supplement of finely ground 1 g of Tetramin fish food [70]. From the culture stock, a subsample of 20 individuals were separated and purged for 5 h to estimate the mean initial biomass of the test worms, in dry weight. These worms were dried at 50 °C (for 24 h) to constant weight and then weighed individually using a Sartorius M3P electrobalance (dl = 1 µg). The mean initial worm biomass was therefore 0.107 ± 0.049 mg dry weight (dw).

The acute toxicity test was performed according to the methodology described by [70] with the metal salt, sodium arsenate dibasic heptahydrate (Na_2_HAsO_4_ × 7H_2_O, Molecular Weight: 312.01 g mol^−1^), to obtain Arsenate (As^5+^), which is the chemical species that typically predominates in well-oxygenated waters and sediments [71]. Briefly, the first step consisted of the preparation of a 1000 mg L^−1^ stock solution using an ACS (American Chemical Society) reagent metal from which serial dilution was carried out for the acute toxicity test, consisting of six increasing nominal tests of the metal salt (96 mg L^−1^, 153 mg L^−1^, 244 mg L^−1^, 390 mg L^−1^, 625 mg l^−1^, and 1000 mg L^−1^). Five experimental replicates were performed for each concentration. A control series with worms and only dechlorinated tap water (no metal) was also included. The test was performed in an incubator at 22 ± 1 °C, in the dark, for 96 h and without aeration or added food. The toxicity test actual concentrations were measured and are given in Table 1.

The test was run in 250 mL glass beakers, pre-washed in acid solution (10% HNO_3_), and with 100 mL of test solution in each beaker. Before placing the worms (a total of 175 individuals) into the beakers, they were distributed in Petri dishes in groups of 5 individuals with dechlorinated water to let them purge their guts during 4–5 h at room temperature [70]. After that period, each group of worms was randomly distributed into the test beakers, and then covered with Parafilm^®^ to prevent water loss. Five individuals of *T. tubifex* were added per beaker. Before adding the oligochaetes to the beakers, a sample of 7 mL of each solution was taken and preserved with 20 µL of nitric acid (70% Baker Instra-Analyzed) for chemical analysis by the SGIKER laboratory (UPV/EHU).

The total exposure period of the toxicity test was 96 h, and every 24 h the mortality and the autotomy were recorded using a stereoscopic microscope. If a worm did not respond in 10 s after disturbance with a bar, it was considered dead [72,73]. The water’s physical (pH, temperature) and chemical characteristics (dissolved oxygen, conductivity) were measured at the middle of the test (48–72 h). Mean values of the replicates for each concentration of pH ranged between 7.76 and 8.47, the conductivity showed a gradient from the less to the highest concentration of 319–1377 µS cm^−1^, and the dissolved oxygen ranged between 7.03 and 7.21 mg L^−1^ (% saturation: 84.0–86.7%).

After 24 h of exposure, one worm from each replicate, if still alive, was removed and stored in 1 mL of RNA*later*™ Solution (Invitrogen, Life Technologies, Carlsbad, CA, USA) at −80 °C. At the end of the 96 h exposure, another worm from each replicate was stored in 1 mL of RNA*later*™ Solution, and the three remaining individuals from each replicate, if still alive, were frozen, freeze-dried, weighed (Sartorius M3P balance), and digested (70% Nitric Acid Baker Instra-Analyzed + 30% H_2_O_2_ Merck Suprapur (Merck, Darmstadt, Germany)) for tissue residue analysis [74].

The metal analysis was conducted using ICP-MS Agilent Serie 7700 (dl = 0.01 µg L^−1^ As) (Agilent, Santa Clara, CA, USA) and ICP-AES (Horiba Jobin Yvon Activa. dl = 0.2 mg L^−1^) by the SGIker Technical Services (UPV/EHU, Leioa, Spain). Mussel Tissue Standard Reference Material (NIST 2976) [75] and TMDA 52.3 [76] were used as reference materials for digested tissues and water samples, respectively. All metal recoveries were within certified values.

### 4.2. Gene Expression Study

#### 4.2.1. Sequencing, De Novo Assembly, and Annotation of a Reference Transcriptome

A second water-only acute toxicity test with arsenic was performed using the same methodology as explained in Section 2.1, and after 96 h, the surviving worms under each condition were stored in RNA*later*^TM^ Solution (Invitrogen, Life Technologies, Carlsbad, CA, USA), for RNAseq. Three cDNA libraries were constructed by the Macrogen company following the Tru-Seq Stranded mRNA (Illumina, San Diego, CA, USA) protocol and sequenced on an Illumina Hi-Seq 4000 using a 100 cycles paired-end protocol. For each sample, total RNA was extracted from pools of three worms using TRIzol Reagent (Invitrogen), following the manufacturer’s instructions. RNA was then treated with DNase I (Invitrogen, Life Technologies, Carlsbad, CA, USA) and extracted using phenol:chloroform:isoamyl alcohol (Fluka Chemie GmbH, Buchs, Switzerland) using 5PRIME Phase Lock Gel Light tubes (Quantabio, Beverly, MA, USA). Purified RNA was resuspended in nuclease-free water, quantified via spectrophotometry at 260 nm using a BioPhotometer (Eppendorf, Hamburg, Germany), and stored at −80 °C.

A reference transcriptome was assembled by integrating the RNA-seq reads from the three libraries obtained. Before assembly, the quality of the sequences was checked using FastQC v0.11.7 [77]; reads with low-quality (Phred value < 33) and adaptor sequences from the raw data were removed using Trimmomatic v0.32 [78]. After trimming, a total of 31,562,136 reads were used to construct the reference transcriptome of this species, with a GC content of 45.43% and a ratio of reads with a Phred quality score of over 30 (Q30) of 95.43%. The filtered reads were thereafter de novo assembled into contigs using Trinity program (version trinityrnaseq_r20140717) [79] and default parameters. All unigenes > 200 bp were searched using BLASTx v2.9.0 against Metazoa with the following protein sequence databases: UNIPROT (v20180116), Kyoto Encyclopedia of Genes and Genomes (KEGG_v20180322) and GO (v20180319) (e-value < 10^−5^), to identify proteins with high sequence similarity, and to assign putative functional annotations. Subsequently, Gene Ontology (GO) annotations of the unigenes were obtained using Blast2GO [80].

#### 4.2.2. Gene Characterisation

Nucleotide sequences for relevant genes relating to cell stress response, energy metabolism, oxidative stress response, and homeostasis maintenance were obtained from our *T. tubifex* transcriptome (Ref. PRJEB50858): *Hsc70* (heat shock cognate 70), *Hsp10* (heat shock protein 10), *Hsp60* (heat shock protein 60), *Hsp83* (heat shock protein 83), *COX1* (cytochrome C oxidase I), *MnSOD* (manganese superoxide dismutase), *CAT* (catalase), *GSR* (glutathione reductase), *CaM* (calmodulin), *UBE2* (ubiquitin-conjugating enzyme), and *RpS13* (40S ribosomal protein S13). SnapGene^®^ v4.3.6 (GSL Biotech LLC, San Diego, CA, USA), BLAST software v2.9.0 [81], and DOG 2.0 software [82] were used in the identification and characterisation of these genes. Sequence alignments were performed using Clustal X Version 2 and MAFFT Version X. SnapGene (GSL Biotech LLC, San Diego, CA, USA). GenBank accession numbers, length, homologous amino acid sequences, and identities are shown in Table A2.

#### 4.2.3. RNA Extraction

The total RNA extraction from *T. tubifex* samples was carried out following the instructions of the TRIzol^®^ commercial kit (Invitrogen, Life Technologies, Carlsbad, CA, USA), consisting of a monophasic solution of phenol and guanidine isothiocyanate suitable for isolating separate fractions of RNA, DNA, and proteins from cells and tissues. The worms were extracted from the freezing vials (containing RNA*later*^TM^) and transferred to 1.5 mL Eppendorf tubes, where they were homogenised in 200 µL of TRIzol^®^ and frozen in dry ice. Later, they were thawed and centrifuged at 12,000 rpm for 10 min at 4 °C, thus allowing the precipitation of cell membranes, polysaccharides, high-molecular-weight DNA, and extracellular material. The supernatant liquid was transferred to another 1.5 mL Eppendorf tube and left for 5 min at room temperature. Then, 40 µL of chloroform (200 µL of chloroform/1 mL of TRIzol^®^) was added and the vials were shaken, letting them rest at room temperature for a further 3 min, after which they were centrifuged again at 12,000 rpm for 15 min at 4 °C.

The aqueous phase containing the RNA was transferred to another 1.5 mL Eppendorf tube, in which the genetic material was precipitated by adding 100 µL of isopropanol (0.5 mL isopropanol/1 mL TRIzol^®^). Finally, the samples were washed 3 times by adding 200 µL of cold 70% ethanol (1 mL ethanol/1 mL TRIzol^®^) and centrifuged at 10,000 rpm for 5 min at 4 °C. The supernatant liquid (ethanol) was decanted, and the precipitated RNA was resuspended in 44.5 µL of nuclease-free water (DEPC-treated water). The samples were thereafter placed in an oven at 55–60 °C for 5 min.

To avoid the presence of DNA remains, the samples were treated with the enzyme, DNase (RNase-free). To the 44.5 µL of sample, 0.5 µL of DNase and 5 µL of buffer were added to obtain a final volume of 50 µL. The mixture was incubated at 37 °C for 30 min. After the incubation period, 50 µL of phenol:chloroform:isoamyl were added to the samples, after which they were centrifuged at 10,000 rpm for 15 min at 4 °C. The aqueous phase (100 µL) was transferred to a Phase Lock Gel column and 50 µL of chloroform were added. The tubes were shaken well by hand and centrifuged at 12,000 rpm for 10 min at 4 °C. The supernatant liquid contained the RNA. It was transferred to another tube and stored at −80 °C.

To determine the concentration of RNA in the samples, the absorption at 260 nm was measured using a spectrophotometer, and the RNA concentration was measured in µg mL^−1^. As indicators of good quality of the RNA samples, A260/280 optical density values between 1.80 and 2.00 and A260/230 values equal to or greater than 1.80 were considered. The integrity of the extracted RNA was checked by running 2 µL of RNA on a 1.5% agarose gel at 60 mV.

#### 4.2.4. Gene Expression Analysis

Five biological replicates with 1 worm per replicate were used for each experimental condition (*n* = 5 worms/condition) in the gene expression analyses. Aliquots containing 7 μg of isolated RNA were reverse-transcribed in a CFX96 Thermal Cycler (Bio-Rad, Hercules, CA, USA) using the iScript Advanced cDNA Synthesis Kit for RT-qPCR (Bio-Rad, USA), according to the kit instructions. The obtained cDNA was conserved at −20 °C and used as a template for subsequent qPCR analyses.

The primers used for the amplification of the selected genes were designed based on the reference *T. tubifex* transcriptome obtained in our laboratory using Primer 3 version 0.4.0 software [83]. The sequences of the primers and the size of the amplified fragments are shown in Table 4. The size of the PCR products was checked in a 1.5% agarose gel at 85 V for 2 h in 1× TBE buffer (Tris-borate-EDTA), stained with ethidium bromide and visualised using Chemigenius 3 (Syngene, Iselin, NJ, USA). The identities of the amplified fragments were verified via Sanger sequencing and BLAST [84]. Amplification efficiencies and correlation coefficients for each primer pair were calculated as described in the Real-Time PCR Applications Guide (Bio-Rad catalogue #170–9799). For all genes, the efficiencies were between 90% and 105% (R^2^ > 0.980).

Real-time quantitative PCR (qPCR) was performed using a QuantStudio 1 Real-Time PCR System (Applied Biosystem, Waltham, MA, USA) with the Power SYBR™ Green PCR Master Mix (Applied Biosystem, Waltham, MA, USA), according to the manufacturer’s protocol. Each qPCR was conducted in a 10 μL mixture containing 1 μL of sample cDNA, 0.6 μL of each primer (10 μM), 4.8 μL of nuclease-free water, and 5 μL of 2× Power SYBR™ Green PCR Master Mix. The qPCR cycling parameters were as follows: 30 s initial denaturation at 95 °C, followed by 40 cycles of 5 s denaturation at 95 °C and 30 s annealing at 58 °C, with a final 30 s for elongation at 60 °C.

Gene encoding of the *28S* ribosomal subunit and GAPDH were used as endogenous reference genes. A fragment of *28S* was amplified using a pair of primers designed for ecotoxicity studies in *C. riparius* [69], and its identity was confirmed by Sanger sequencing (STABvida company, Caparica, Portugal). The statistical validation of the stability of the reference genes was performed by means of real-time PCR QuantStudio 1 (Applied Biosystems, Waltham, MA, USA), using an iterative test for pairwise variation, according to Vandesompele [85]. The 2^−ΔΔCt^ method was used to analyse relative changes in gene expression using the Quantstudio™ Design & Analysis Software, Version 2.7 (Thermo Fisher Scientific, Waltham, MA, USA) For each experimental condition studied, five biological replicates were analysed, and three technical replicates were carried out. Gene expression results were normalised to the control values for subsequent analysis.

### 4.3. Statistical Analyses

The NOEC (no-observed effect concentration) and the LOEC (lowest-observed effect concentration) were identified using the Kruskal–Wallis non-parametric test and Dunn’s test, as a post hoc pairwise comparison test (using IBM^®^SPSS^®^ Statistics 24 software) [86].

Lethal and median effect concentrations and their 95% confidence limits (LC_50_, EC_50_, LR_50_, and ER_50_, 95% CL) were estimated for autotomy and mortality at 24, 48, 72, and 96 h of exposure adjusting data to the Probit model (using IBM^®^SPSS^®^ Statistics 24) [83]. In a few cases (data from 72 h and 96 h of exposure), data were smoothed. When it was not possible to adjust data to the Probit model for calculating the LC_50_, EC_50_, LR_50_, and ER_50_, the Trimmed Spearman–Karber method was used [87].

For gene expression study, statistical analyses were performed using R. 3.4.3 software [88]. Mean and median were calculated, respectively, as the average and the middle of a data set, while the standard deviation represented the square root of the variance. Regarding the survival studies, the Student T test was used to check the statistically significant differences (*p* < 0.05) between the different experimental conditions. For transcriptional analyses, the normality and homoscedasticity of the data were checked using the Shapiro–Wilk and Levene tests, respectively. Normal and homoscedastic data were analysed via ANOVA followed by Bonferroni’s post hoc test. Otherwise, differences in transcript levels were evaluated using the nonparametric Kruskal–Wallis Test followed by Mann–Whitney–Wilcoxon post hoc test. *p*-value < 0.05 and *p*-value < 0.1 were used as cut-offs for statistical significance.

## 5. Conclusions

The assembled transcriptome of *T. tubifex* is a valuable resource for the identification of potential molecular biomarkers to deepen the knowledge of the physiological effects on this species caused by exposure to heavy metals. Eleven novel genes related to cell stress response, energy production, oxidative stress, and cell homeostasis were identified, de novo demonstrating differential expression in *T. tubifex* after having been exposed to different concentrations of arsenic, an efficient method to better understand the physiological response of these worms to this type of heavy metal in sediments. Arsenic resulted in the repression of genes related to mitochondrial energy metabolism, ribosome biosynthesis, cell homeostasis, and ubiquitin–proteasome system. The induction of the response to oxidative stress and the cell stress response mediated by HSPs was also remarkable. We have shown that the estimation of NOEC, LOEC, LC_50_, EC_50_, LR_50_, and ER_50_ for As has proven to be a highly effective tool in assessing the As acute toxicity of the aquatic oligochaete, *T. tubifex*. Together, the combination of classical and molecular tools could lead to more robust and efficient environmental risk assessment, and also lead to a better understanding of the risks that heavy metals may pose for aquatic worms.

## Figures and Tables

**Table 1:** Total mortality and autotomy data at 24, 48, 72, and 96 h of actual As exposure in the *T. tubifex* water-only toxicity test. Control value refers to As 0.00 mg L^−1^.

As (mg L^−1^)	Mortality				Autotomy			
	24 h	48 h	72 h	96 h	24 h	48 h	72 h	96 h
0.00	0	1	1	1	0	0	0	0
38	0	0	0	0	0	0	0	0
63	0	0	0	0	2	2	2	2
100	0	2	6	11	2	8	12	12
159	4	7	11	13	6	11	13	15
250	2	9	18	18	8	18	18	18
407	12	14	15	16	13	15	16	16

**Table 2:** NOEC, LOEC, and EC_50_ in *T. tubifex* (in mg L^−1^) with their 95% confidence interval (95% CI), related to mortality and autotomy after 24, 48, 72, and 96 h of exposure to As in a water-only toxicity test. * Trimmed Spearman–Karber method. Abbreviations: *p*, test probability of rejecting H_0_; ns, not significant.

	Mortality				Autotomy			
	24 h	48 h	72 h	96 h	24 h	48 h	72 h	96 h
**NOEC**	250	-	159	159	-	159	159	159
**LOEC**	407	ns	250	250	ns	250	250	250
**(*p* value)**	(0.041)	-	(0.019)	(0.028)	-	(0.013)	(0.011)	(0.015)
**LC50/EC_50_**	>407	260	163	137 *	387	149	126	120
**95% CI**	-	210–349	135–199	136–137	280–718	120–188	101–155	96–148

**Table 3:** Effective tissue residues (in µg g^−1^ dw) in *T. tubifex*, LR_50_ (mortality), and ER_50_ (autotomy) and their 95% confidence interval (95% CI) related to mortality and autotomy, after 24, 48, 72, and 96 h of exposure to As in a water-only toxicity test. * Trimmed Spearman–Karber method.

	Mortality				Autotomy			
	24 h	48 h	72 h	96 h	24 h	48 h	72 h	96 h
**LR_50_/ER_50_**	1072	-	679 *	578 *	1019	634 *	555 *	479 *
**95% CI**	891–1502	-	671–687	574–582	795–1623	630–638	553–557	477–481

**Table 4:** Primers used for cDNA sequencing and real-time qPCR of genes studied in *T. tubifex*. Forward (F) and reverse (R) sequences, length of amplified fragments, and origin of primers when corresponding.

Gene	Primer Sequence (5′–3′)	Fragment Size (bp)
*Hsc70*	**F** ATGGACAAGCCGGCCATCCACG **R** GAGGGACAGCGGAGCAACGT	228
*Hsp10*	**F** AAAGGTGCTCGAAGCGACAG **R** ACGACTGGAATTTGCCGAGA	191
*Hsp60*	**F** GGTCCAAGACATCGCACACA **R** GGCTTCCATCACACCTCGTC	151
*Hsp83*	**F** GAGCAGATGGAGGACGGAGA **R** CGAATCTTGTCGAGGGCATC	152
*COX1*	**F** CGGGTGTATGCTTAGCAAACTCA **R** CCGAATACTGCCCCCATTCT	103
*Cat*	**F** GTGCTGAACCGTAGCCCAAA **R** ACGAGAACAGACGACCCTGAAG	124
*GSR*	**F** ACCGTTGTGTTCAGCCATCC **R** TTCTTTTGTGTCATTGCGTGGT	137
*MnSOD*	**F** TGCCGAAGCACAGGCTAAA **R** CTCAAGCGAACCAAAGTCACG	184
*CaM*	**F** AAGGAACTGGGGACCGTGAT **R** TCAGGAACTCGGGAAAGTCTATTG	121
*RpS13*	**F** TTGGCGTTATTCTGCGTGACT **R** TTGTCCTTGCGGTTCCTCTC	178
*UBE2*	**F** CGTCTGCTTGTGGTGGTGAC **R** TCGTTGTCCATCTCGTCGTAATAAT	118
*Gapdh*	**F** GGCAAGCTGACTGGAATGGC **R** TGCCGCTTTGATTTGGTCGT	108
*28S*	**F** TTCGCGACCTCAACTCATGT **R** CCGCATTCAAGCTGGACTTA	220

## Data Availability

Data are available within the article.

## Appendix A

**Table A1:** Mean values of the temperature, pH, conductivity, and dissolved oxygen in the water column of toxicity test beakers.

Arsenic (mg L^−1^)	Temperature (°C)	pH	Conductivity (µS cm^−1^)	Dissolved Oxygen (% SAT)	Dissolved Oxygen (mg L^−1^)
0	23.6	7.76	319	85.8	7.18
38	23.5	8.12	415	85.2	7.12
63	23.5	8.25	477	85.4	7.09
100	23.7	8.28	577	86.1	7.19
159	23.5	8.31	733	84.0	7.03
250	23.6	8.40	979	85.2	7.10
407	23.7	8.47	1377	86.7	7.21

**Table A2:** De novo characterised *T. tubifex* genes related to cell stress response, energy metabolism, detoxification and oxidative stress response, and homeostasis maintenance. Gene name, ORF, and protein lengths as well as the percentage of identity likeness to the closest species on databases are provided. * Incomplete protein.

Gene	Species	Accession Number	Length (aa)	Identity (%)
*Hsc70*	*Tubifex tubifex*	PP213149	482	-
	*Biomphalaria glabrata*	XP_013082114.1	649	89
	*Sacostrea echinata*	XP_061181462.1	659	89
*Hsp10*	*Tubifex tubifex*	PP213150	101	-
	*Crassostrea gigas*	XP_011456448.1	101	76
	*Physella acuta*	XP_059140133.1	101	75
*Hsp60*	*Tubifex tubifex*	PP213151	588	-
	*Helobdella robusta*	XP_009028388.1	584	78
	*Eisenia fetida*	WRM33298.1	586	80
*Hsp83*	*Tubifex tubifex*	PP213152	280 *	-
	*Halyomorpha halys*	XP_014284945.1	723	87
	*Arma chinensis*	WGV41501.1	723	82
*COX1*	*Tubifex tubifex*	PP212898	511	-
	*Limnodrilus hoffmeisteri*	QWT71538.1	512	86
	*Nais communis*	QUT13265.1	512	81
*Cat*	*Tubifex tubifex*	PP213153	509	-
	*Eisenia fetida*	AEO50756.2	505	74
	*Haliotis madaka*	ALU63753.1	509	75
*GSR*	*Tubifex tubifex*	PP213154	456	-
	*Haliotis fulgens*	AXR98615.1	452	65
	*Pomacea canaliculata*	XP_025078581.1	452	65
*MnSOD*	*Tubifex tubifex*	PP213155	226	-
	*Eisenia andrei*	AMZ80133.2	225	69
	*Zootermopsis nevadensis*	XP_021916368.1	225	69
*CaM*	*Tubifex tubifex*	PP213156	149	-
	*Lumbricus rubellus*	Q9GRJ1.3	149	100
	*Pinctada fucata*	AAQ20043.1	149	99
*RpS13*	*Tubifex tubifex*	PP213157	151	-
	*Arenicola marina*	ABW23143.1	151	91
	*Lumbricus rubellus*	O77303.3	151	88
*UBE2*	*Tubifex tubifex*	PP213158	250 *	-
	*Branchiostoma floridae*	XP_035678611.1	342	65
	*Lingula anatina*	XP_013421956.1	319	63
*Gapdh*	*Tubifex tubifex*	PP213159	336	-
	*Enchytraeus albidus*	AOR07119.1	336	81
	*Mesenchytraeus hydrius*	AOR07125.1	336	78
